# Interoception, insula, and agency: a predictive coding account of aphantasia

**DOI:** 10.3389/fpsyg.2025.1564251

**Published:** 2025-05-07

**Authors:** Juha Silvanto

**Affiliations:** ^1^Centre for Cognitive and Brain Sciences, University of Macau, Taipa, Macau SAR, China; ^2^School of Psychology, Faculty of Health and Medical Sciences, University of Surrey, Guildford, United Kingdom

**Keywords:** aphantasia, mental imagery, interoception, insula, agency, predictive coding

## Introduction

Aphantasia (the inability to consciously experience mental imagery) has emerged as one of the most intriguing phenomena in cognitive psychology and neuroscience (see e.g., Nanay, 2021; Zeman, 2024). Recently, we proposed an interoceptive model of aphantasia, suggesting that the condition arises from suboptimal processing in the insula (Silvanto and Nagai, 2025a). In this model, interoception contributes to imagery on two interrelated components: (1) the integration of interoceptive input with sensory information, which anchors imagined content in the bodily self and gives rise to a sense of embodiment, and (2) sense of agency, which enables the voluntary initiation and control of mental content. Here, we apply a predictive coding framework to aphantasia and propose that reduced interoceptive precision disrupts the brain's ability to generate high-gain top-down predictions about imagined content. As a result, predictive signals from the prefrontal cortex fail to sufficiently activate parietal and visual regions, preventing the formation of coherent sensory representations and leading to the failure of mental imagery to reach conscious awareness.

## Sense of agency and mental imagery

The embodied nature of imagery (Muraki et al., 2023), and the importance of integrating interoceptive input with sensory representations, have been discussed in detail (Silvanto and Nagai, 2025a). In contrast, the mechanisms through which interoception contributes to the sense of agency in mental imagery have been less explored. The sense of self is composed of two main components: body ownership (the feeling that your body belongs to you; e.g., Tsakiris et al., 2010) and sense of agency (the feeling that you are in control of your own actions; Gallagher, 2007; Haggard, 2017). These experiences emerge from the integration of information from within the body (interoception), the external environment, and intentional actions (e.g., Blanke et al., 2015; Haggard, 2008). Interoception connects bodily sensations to intentional movements, grounding the sense of agency (Tsakiris et al., 2010). The experience of agency emerges from anticipatory mechanisms within the motor system. The brain generates an internal duplicate of a motor instruction, referred to as an efference copy, which forecasts forthcoming bodily states and associated sensory feedback (Frith, 1992). This prediction is then evaluated against both the intended goal and the actual outcome, facilitating rapid motor adjustments and reinforcing the feeling of control. Within the comparator framework, when expected and actual results match, the sense of agency is affirmed; if discrepancies arise, the sensation of control diminishes. This mechanism aligns with predictive coding models, in which the brain continuously updates its predictions about sensory input to minimize error (Seth, 2015).

The importance of agency in the present context is reflected in the fact that, while individuals with aphantasia are unable to generate voluntary mental images, they can still experience involuntary imagery, such as dreams. For instance, Zeman et al. (2015) reported that 17 of 21 individuals with weak or absent visual imagery experienced visual dreams. In a larger study by Zeman et al. (2020) found that 63.4% of 2,000 participants with aphantasia reported visual dreaming. While dreams are often less vivid and frequent in aphantasia, their preservation, despite the absence of voluntary imagery, suggests that volitional control is a primary factor disrupted in aphantasia (but see Krempel and Monzel, 2024, for an alternative view). During sleep, interoceptive input is suppressed (Wei and Van Someren, 2020) and interoceptive feelings during dreaming are uncommon (Mazza et al., 2012). The (at least partial) preservation of dreaming in aphantasia highlights the distinction of interoceptive involvement in voluntary vs. involuntary imagery, with the former affected of aphantasia.

There is some initial evidence linking sense of agency to aphantasia (Silvanto and Nagai, 2025b). Individuals with stronger sense of general agency (a measure reflecting agency across various situations and circumstances) were more likely to report experiencing mental imagery (as measured with the Vividness of Visual Imagery Questionnaire). This indicates that a general bias toward interpreting events as self-generated facilitates mental imagery, particularly in individuals at the lower end of imagery spectrum. However, it is important to note that this study relied entirely on self-report measures. Future experiments should complement these findings with behavioral paradigms (e.g., intentional binding) and neuroimaging techniques to clarify the underlying neural mechanisms.

## Neural basis of agency

A large network of brain regions has been linked to the sense of agency, including the premotor cortex, supplementary motor areas (SMA and pre-SMA), insula, posterior parietal cortex (PPC), and the cerebellum (e.g., Haggard, 2017). One key challenge has been to distinguish neural activity related to sensory input from that associated with subjective experiences of the bodily self. Harduf et al. (2023) addressed this challenge using the rubber hand illusion, a phenomenon where individuals perceive a fake rubber hand as part of their body (Ehrsson et al., 2004). They found that while multisensory integration in occipital and fronto-parietal regions encoded sensory inputs, participants who experienced heightened agency during the illusion exhibited stronger connectivity between the insula, left occipital cortex, and somatosensory regions. Greater illusory body ownership was associated with increased connectivity between the insula and somatosensory cortices, underscoring the insula's critical role in integrating sensory and interoceptive signals.

The insula is thought to support agency by integrating interoceptive and sensory inputs to align predicted and actual bodily states. It plays a central role in integrating autonomic, visceral, and somatic information to support emotional awareness and self-representation (Critchley et al., 2005). The insula is also part of the salience network, a system important for self-awareness and evaluating the importance of internal and external stimuli (Uddin, 2015). By facilitating interoceptive awareness and anchoring internal states within the broader framework of self-referential cognition, the insula is key to an embodied sense of self. From a predictive coding perspective, the sense of agency arises as the brain generates top-down predictions about the sensory consequences of actions and refines them based on interoceptive and sensory feedback (Seth et al., 2012). The insula contributes by integrating interoceptive priors, which provide a physiological foundation for agency by aligning internal bodily states with motor and sensory predictions. The anterior cingulate cortex (ACC) further refines these predictions by monitoring prediction errors, ensuring coherence between expected and actual outcomes.

## Predictive coding model of aphantasia

Predictive coding offers a broad explanatory framework for conscious experience by proposing that the brain constructs reality by integrating exteroceptive and interoceptive priors to minimize prediction error (Seth et al., 2012; Seth, 2015). Conscious experiences arise when top-down expectations are precise enough to successfully predict incoming input, thereby minimizing error. When errors are too large, perception becomes unstable or fragmented; when too small, experience becomes overly rigid, dominated by inflexible priors. In this framework, mental imagery involves the generation of high-level predictions that are both precise and embodied, even in the absence of external input. Interoceptive priors provide affective and bodily grounding, while motor-based forward models (internal predictions of the sensory consequences of self-generated actions) confer a sense of agency; both are essential for internally generated simulations to become conscious.

Mental imagery relies on internally generated top-down predictions to activate sensory representations in the absence of external input. When imagery is voluntarily initiated, prefrontal regions generate predictions which activate sensory areas such as the visual cortex. For these predictions to give rise to imagery, they must be assigned sufficient gain; a process modulated by the insula, which evaluates the relevance and reliability of internal bodily states. The insula estimates the precision of interoceptive input which then modulates the gain of prefrontal, imagery-related top-down predictions, thereby influencing whether internally generated representations are treated as reliable and permitted to shape sensory inference. This gain modulation depends on two functions supported by the insula that are essential to imagery: agency, the capacity to initiate and control mental content, and embodiment, the integration of imagined content with bodily self-awareness. When either of these components is weakened, imagery becomes unstable and fails to reach conscious awareness.

**Figure 1:**
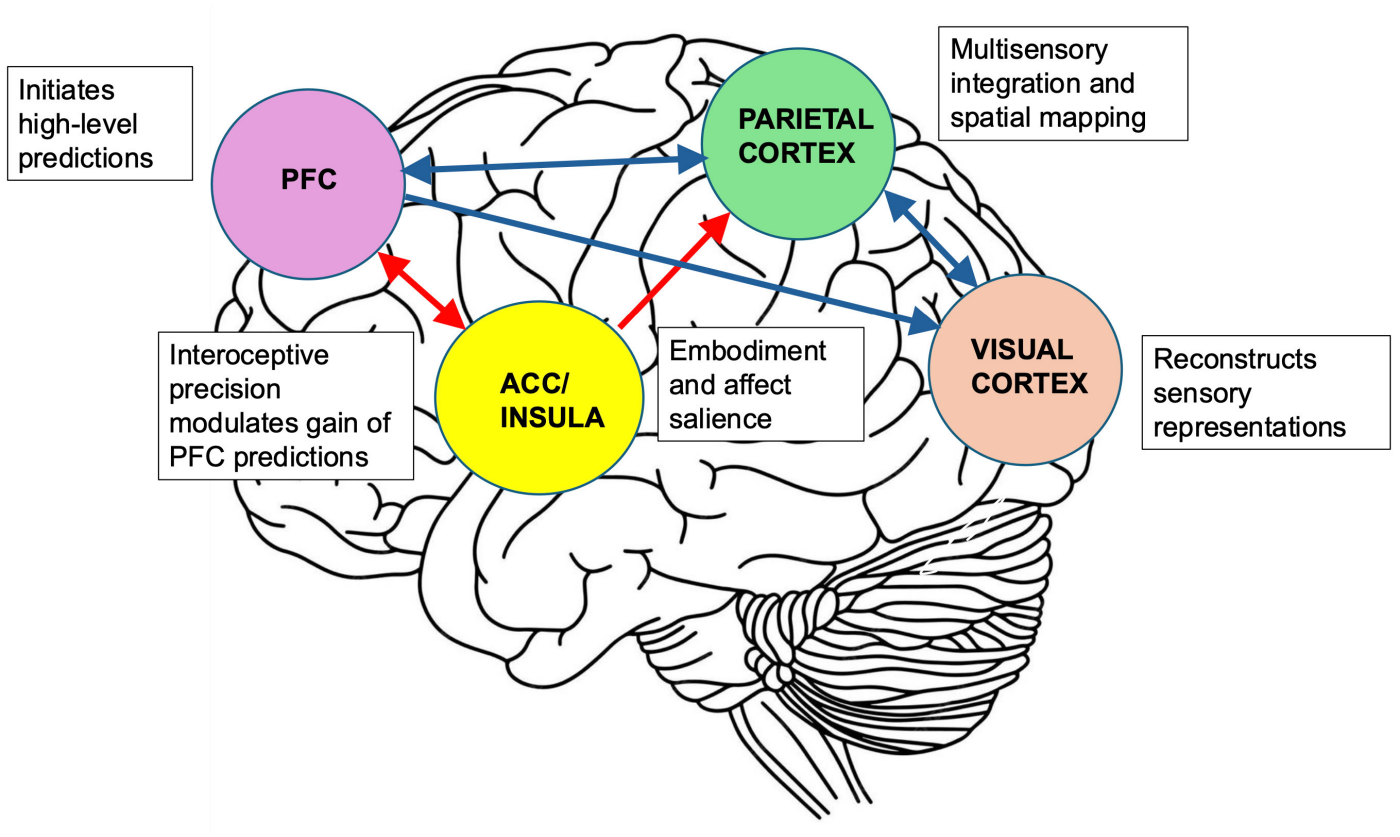
Predictive coding framework for mental imagery and its disruption in aphantasia. The prefrontal cortex (PFC) initiates high-level predictions related to the content of mental imagery. These influence the parietal cortex, which refines sensory content, and the visual cortex, which reconstructs perceptual representations. The insula and anterior cingulate cortex (ACC) estimate the precision of interoceptive signals and modulate the gain of PFC-generated predictions. They also modulate the parietal cortex by embedding perceptual priors with bodily and affective relevance. In aphantasia, disrupted insula–ACC function diminishes interoceptive precision, weakening both the experience of agency and the integration of bodily signals. As a result, top-down predictions from the prefrontal cortex to sensory regions receive insufficient gain, leading to diminished activation of these regions. This prevents the conscious experience of mental imagery. In addition, impaired insula–parietal interactions reduce the affective salience and embodied quality of imagery content, further contributing to its failure to reach awareness. Red arrows in the figure indicate disrupted pathways; blue arrows represent intact predictive hierarchies. Bidirectional arrows depict reciprocal coding loops; unidirectional arrows reflect dominant top-down flow.

In this framework, agency depends on the brain's ability to predict the sensory consequences of self-generated mental actions, analogous to motor control systems in which efference copies anticipate the outcomes of movement (Frith, 1992). When interoceptive precision is low, the insula downregulates the gain of these top-down signals, leading to diminished confidence in the initiation of mental acts. This same mechanism applies to embodiment. By aligning interoceptive information with exteroceptive input and higher-order predictions, the insula contributes to the embodied and self-referential nature of mental imagery. Through its interactions with parietal regions, it further modulates the affective salience and bodily relevance of intermediate-level predictions. In this view, aphantasia reflects a failure to generate internally consistent predictions that are sufficiently grounded in bodily and volitional signals (see Figure 1).

In perceptual processing, continuous bottom-up sensory input helps to constrain and update predictive models by providing real-time information about the external environment. This input stabilizes perceptual inference, even in the presence of imprecise or underspecified top-down predictions. In contrast, mental imagery lacks such external sensory input and must rely exclusively on internally generated predictions; under these conditions, interoceptive input has a heightened role. When interoceptive signals are assigned low precision, top-down predictions lack sufficient gain to overcome prediction error and are downweighted; consequently, internally generated simulations fail to reach conscious experience. This framework helps to explain why disruptions to interoceptive processing may disproportionately impair mental imagery; whereas perception can be corrected and stabilized through external feedback, imagery depends entirely on the precision of top-down models.

## Impaired cross-modal processing in aphantasia

Consistent with this model, there is evidence for impaired top-down connectivity from prefrontal to visual cortices in aphantasia (Milton et al., 2021; Liu et al., 2025; Spagna et al., 2021). Moreover, these impairments appear to extend beyond visual imagery, affecting cross-modal processes that rely on top-down feedback, such as the integration of auditory and visual information. Montabes de la Cruz et al. (2024) found that, in aphantasia, decoding of auditory scenes was preserved in auditory and multisensory cortices but reduced in early visual cortex, indicating that integration-based deficits in aphantasia apply not only to visual imagery but also to the broader coordination of information across sensory modalities. These findings can be readily accommodated within the insula-based model of aphantasia (cf. Silvanto and Nagai, 2025a) by extending it to include impaired integration of not only interoceptive and exteroceptive signals, but also within exteroceptive modalities themselves. The role of the insula as a central hub for multisensory integration is well established; it receives input from the thalamus and multiple cortical regions, allowing it to combine information from external senses such as vision, audition, touch, taste, and smell (e.g., Gogolla, 2017; Craig, 2009; Garfinkel and Critchley, 2016) thus supporting crossmodal binding across domains. However, imagery is more dependent on its modulatory function because it lacks bottom-up sensory input to stabilize cross-modal predictions. In contrast, perceptual systems can rely on externally driven prediction errors to align sensory modalities. As a result, when insular function is compromised, imagery-related processes are more likely be severly affected.

## Discussion

In the interoceptive theory of imagery, aphantasia arises from disrupted processing in the insula (Silvanto and Nagai, 2025a). Two core components are affected: agency, the capacity for voluntary control over actions and mental states; and embodiment, the sense that mental content belongs to the self, arising from impaired integration of internal bodily states with sensory information. Predictive coding provides a mechanistic framework for understanding the role of the insula in aphantasia, specifically its function in determining the precision of interoceptive signals and regulating the gain of top-down predictions originating from the prefrontal cortex, processes that are critical for generating consciously accessible mental imagery. It is important to acknowledge, however, that this model remains a theoretical proposal, as direct empirical evidence linking interoception and imagery in aphantasia is currently limited (but see Nagai et al., 2025, for some evidence).
